# Supplementary material for: Host and viral determinants for efficient SARS-CoV-2 infection of the human lung
Source: Nat Commun. 2021 Jan 8;12:134. doi: 10.1038/s41467-020-20457-w (PMC7794309; doi:10.1038/s41467-020-20457-w)
Supplement: Supplementary file 1 — Supplementary Information [file 41467_2020_20457_MOESM1_ESM.pdf]

Host and viral determinants for efficient SARS-CoV-2 infection of the human lung

Hin Chu[1,2,*], Bingjie Hu[2,*], Xiner Huang[2,*], Yue Chai[2,*], Dongyan Zhou[2], Yixin Wang[2], Huiping Shuai[2], Dong Yang[2], Yuxin Hou[2], Xi Zhang[2], Terrence Tsz-Tai Yuen[2], Jian-Piao Cai[2], Anna Jinxia Zhang[1,2], Jie Zhou[1,2], Shuofeng Yuan[1,2], Kelvin Kai-Wang To[1,2,3,4], Ivy Hau-Yee Chan[5], Ko-Yung Sit[5], Dominic Chi-Chung Foo[5], Ian Yu-Hong Wong[5], Ada Tsui-Lin Ng[5], Tan To Cheung[5], Simon Ying-Kit Law[5], Wing-Kuk Au[5], Melinda A. Brindley[6], Zhiwei Chen[1,2,3], Kin-Hang Kok[1,2,3], Jasper Fuk-Woo Chan[1,2,3,4,#], Kwok-Yung Yuen[1,2,3,4,#]

Supplementary Figure and Figure legends 1-7

Supplementary Table 1

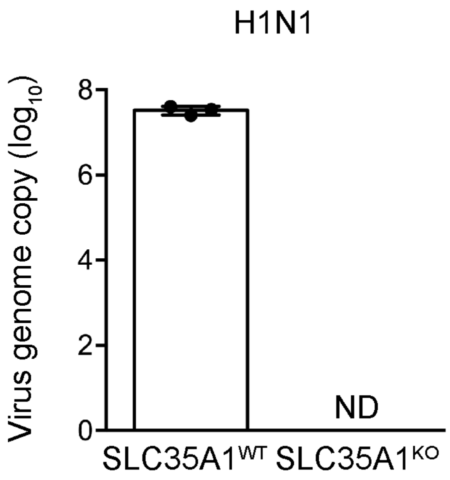

Supplementary Figure 1. Solute carrier family 35 member A1 (SLC35A1) knockout HEK293 cells are resistant to influenza virus infection. The SLC35A1^{WT} and SLC35A1^{KO} 293 cells were infected with H1N1 at 0.1MOI for 1h at 37 °C. Cell lysates were collected at 24hpi and lysed for qRT-PCR analysis (n=3). Data represented mean and standard deviations from the indicated number of biological repeats. Source data are provided as a Source Data file.

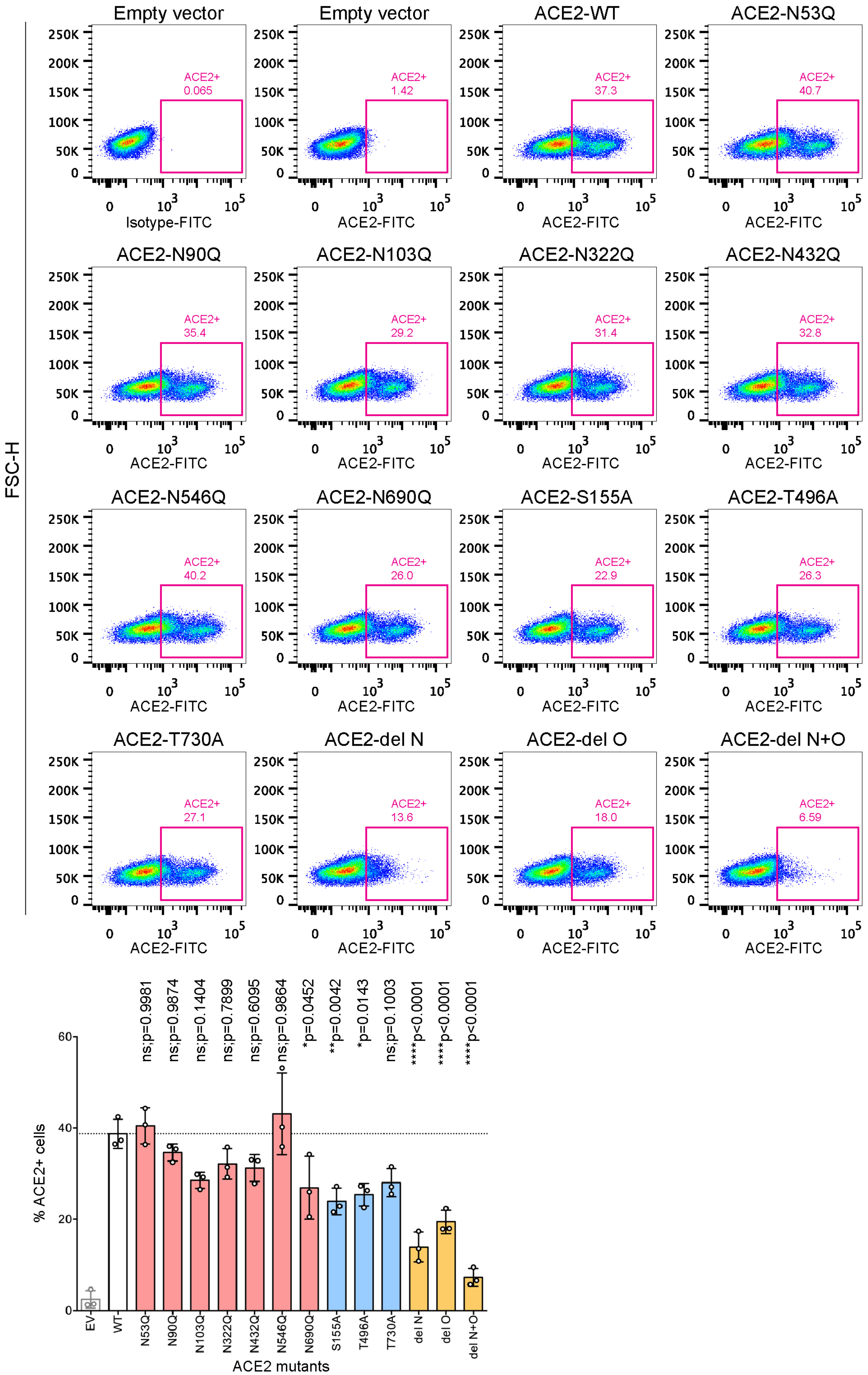

FSC-H
Empty vector
ACE2+ 0.065
Isotype-FITC
Empty vector
ACE2+ 1.42
ACE2-FITC
ACE2-WT
ACE2+ 37.3
ACE2-N53Q
ACE2+ 40.7
ACE2-N90Q
ACE2+ 35.4
ACE2-N103Q
ACE2+ 29.2
ACE2-N322Q
ACE2+ 31.4
ACE2-N432Q
ACE2+ 32.8
ACE2-N546Q
ACE2+ 40.2
ACE2-N690Q
ACE2+ 26.0
ACE2-S155A
ACE2+ 22.9
ACE2-T496A
ACE2+ 26.3
ACE2-T730A
ACE2+ 27.1
ACE2-del N
ACE2+ 13.6
ACE2-del O
ACE2+ 18.0
ACE2-del N+O
ACE2+ 6.59
250K
200K
150K
100K
50K
0
0
10^3
10^5
% ACE2+ cells
ACE2 mutants
EV
WT
N53Q
N90Q
N103Q
N322Q
N432Q
N546Q
N690Q
S155A
T496A
T730A
del N
del O
del N+O
ns;p=0.9981
ns;p=0.9874
ns;p=0.1404
ns;p=0.7899
ns;p=0.6095
ns;p=0.9864
*p=0.0452
**p=0.0042
*p=0.0143
ns;p=0.1003
****p<0.0001
****p<0.0001
****p<0.0001

Supplementary Figure 2. Cell surface expression of the ACE2 glycosylation mutants.

BHK21 cells were transfected with the ACE2 glycosylation mutants. At 24 hours post transfection, the cells were harvested for flow cytometry analysis of cell surface ACE2 expression (n=3). Data represented mean and standard deviations from the indicated number of biological repeats. Statistical analysis was performed with one-way ANOVA. * represented $p < 0.05$, ** represented $p < 0.01$, **** represented $p<0.0001$. ns = not significant. Source data are provided as a Source Data file.

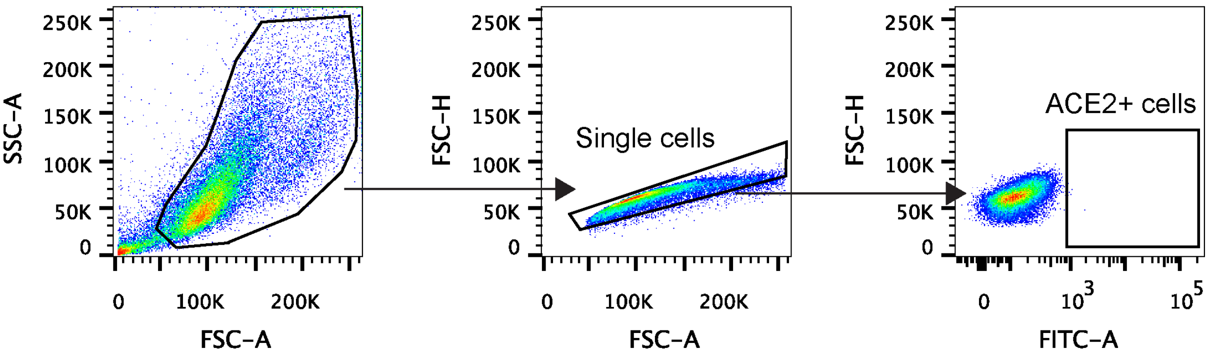

Supplementary Figure 3. Gating strategy for the flow cytometry panels presented in Figure S2. The main BHK21 cell population was gated with SSC-A vs FSC-A. The selected cells were gated with FSC-H vs FSC-A for single cells. From that, ACE2 positive cells were gated with FSC-H vs FITC-A. BHK21 cells labeled with isotype control and secondary antibody were used as the control for gating.

Genome	Position	Nucleotide	Gene	Amino acid
SARS-CoV-2 HKU001a	802	(GGG)	NSP2	G179
SARS-CoV-2 HKU001a S_1/S_2mut	802	(GGA)	NSP2	G179
SARS-CoV-2 HKU001a	5947	(CCC)	NSP3	P1076
SARS-CoV-2 HKU001a S_1/S_2mut	5947	(CCT)	NSP3	P1076
SARS-CoV-2 HKU001a	11074	(TTT)	NSP6	F34
SARS-CoV-2 HKU001a S_1/S_2mut	11074	(TTC)	NSP6	F34
SARS-CoV-2 HKU001a	18314	(GAG)	NSP14	E92
SARS-CoV-2 HKU001a S_1/S_2mut	18314	(GGG)	NSP14	G92
SARS-CoV-2 HKU001a	23597-23626	(AAT TCT CCT CGG CGG GCA CGT AGT GTA GCT)	S	NSPRRARSVA (688-697)
SARS-CoV-2 HKU001a S_1/S_2mut	23597-23626	(Deletion)	S	Deletion (688-697)
SARS-CoV-2 HKU001a	29051	(CAA)	N	Q260
SARS-CoV-2 HKU001a S_1/S_2mut	29051	(GAA)	N	E260
SARS-CoV-2 HKU001a	29386	(GAA)	N	E371
SARS-CoV-2 HKU001a S_1/S_2mut	29386	(GAC)	N	D371

Supplementary Figure 4. Sequence alignment between SARS-CoV-2 HKU-001a and SARS-CoV-2 HKU001a S_1/S_2mut. The nucleotide and amino acid sequences of SARS-CoV-2 HKU001a (MT230904) and SARS-CoV-2 HKU001a S_1/S_2mut (MT621560) were compared. Amino acid substitutions of the culture strain were labeled in red.

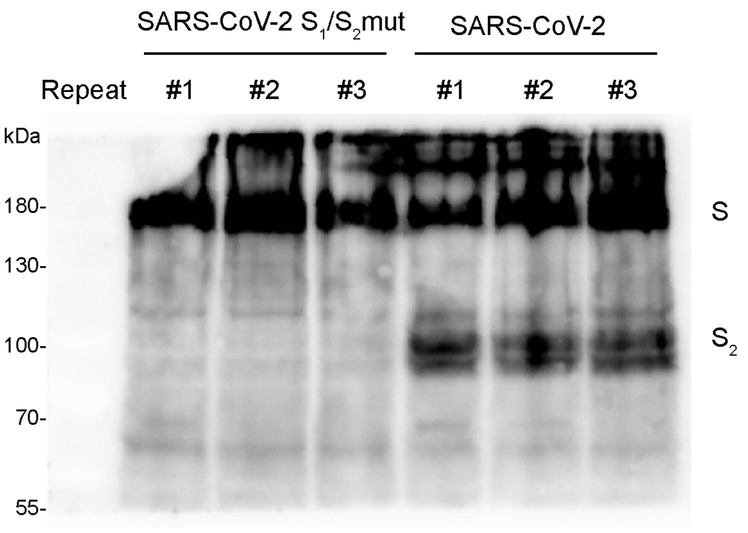

Supplementary Figure 5. Cleavage of SARS-CoV-2 spike and SARS-CoV-2 S_1/S_2mut spike. VeroE6 cells were infected with SARS-CoV-2 or SARS-CoV-2 S_1/S_2mut. At 24 hours, the cell lysates were lysed for Western blot analysis of spike cleavage. Three biological repeats for each virus were shown. Source data are provided as a Source Data file.

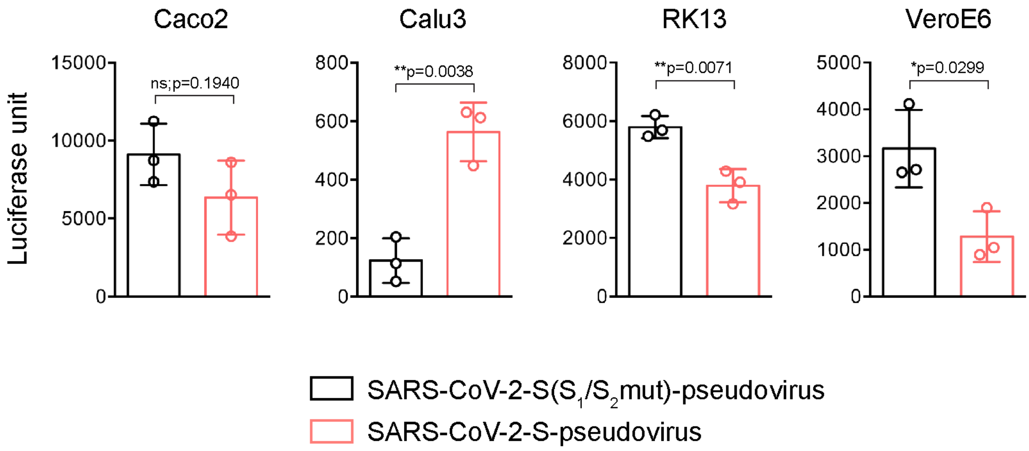

Supplementary Figure 6. SARS-CoV-2-S-pseudovirus or SARS-CoV-2-S(S_1/S_2mut)-pseudovirus entry in Caco2, Calu3, RK13, and VeroE6 cells. Caco2, Calu3, RK13, and VeroE6 cells were inoculated with SARS-CoV-2-S-pseudoviruses or SARS-CoV-2-S(S1/S2mut)-pseudoviruses. At 24 hours post inoculation, the cells were harvested and pseudovirus entry was determined by measuring the luciferase signal (n=3). Data represented mean and standard deviations from the indicated number of biological repeats. Statistical significance between groups was determined with two-sided unpaired Student's t-test. * represented $p < 0.05$, ** represented $p < 0.01$. ns = not significant. Source data are provided as a Source Data file.

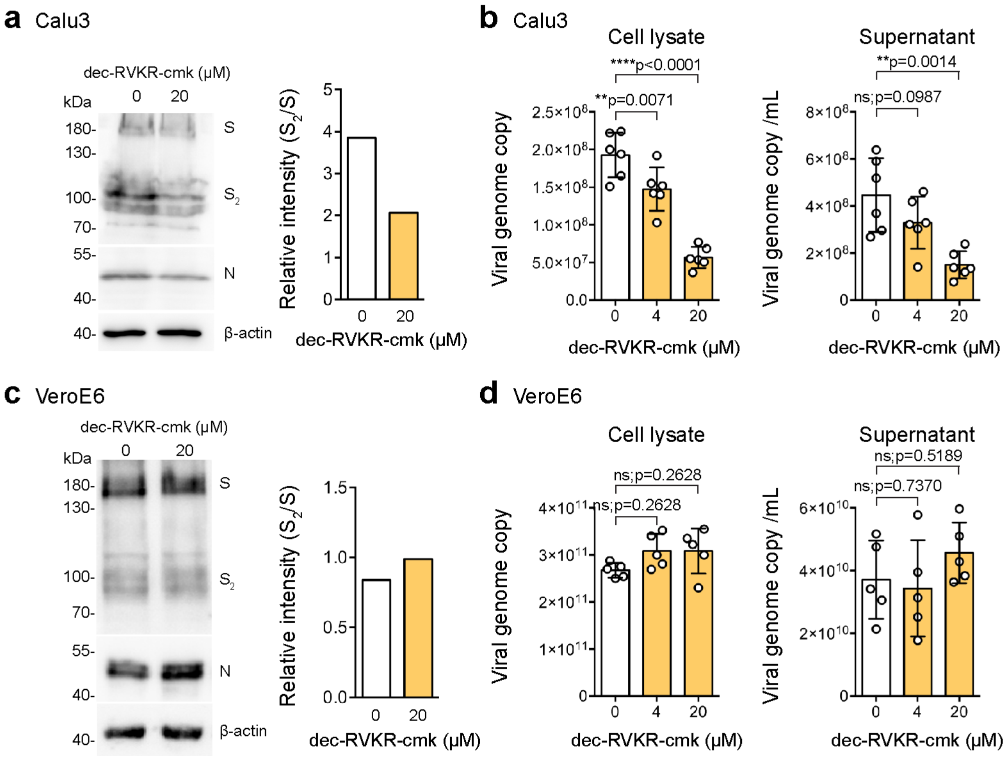

Supplementary Figure 7. Differential role of furin in Calu3 and VeroE6 cells during SARS-CoV-2 infection. a Calu3 cells were infected with SARS-CoV-2 with or without 20µM dec-RVKR-cmk. Spike cleavage was visualized with Western blots. **b** Calu3 cells were infected with SARS-CoV-2 with 0, 4, or 20µM dec-RVKR-cmk. Cell lysate and supernatant samples were harvested at 24hpi for qRT-PCR analysis of virus replication (n=6). **c** VeroE6 cells were infected with SARS-CoV-2 with or without 20µM dec-RVKR-cmk. Spike cleavage was visualized with Western blots. **d** VeroE6 cells were infected with SARS-CoV-2 with 0, 4, or 20µM dec-RVKR-cmk. Cell lysate and supernatant samples were harvested at 24hpi for qRT-PCR analysis of virus replication (n=5). Data represented mean and standard deviations from the indicated number of biological repeats. The experiment in **a** and **c** was repeated three times independently with similar results. Statistical significance between groups was determined with one-way ANOVA. ** represented $p < 0.01$, **** represented $p < 0.0001$. ns = not significant. Source data are provided as a Source Data file.

Supplementary Table 1. Primer list.

Species	Gene	Sequence
SARS-CoV-2	RdRp	Forward 5’- CGCATACAGTCTTRCAGGCT -3’<br>Reverse 5’- GTGTGATGTTGAWATGACATGGTC -3’<br>Probe 5’- FAM-TTAAGATGTGGTGCTTGCATACGTAGAC-lABkFQ -3’
SARS-CoV	RdRp	Forward 5’- CGCATACAGTCTTRCAGGCT -3’<br>Reverse 5’- GTGTGATGTTGAWATGACATGGTC -3’<br>Probe 5′-Cy5-CTTCGTTGCGGTGCCTGTATTAGG-IAbRQSp-3′
MERS-CoV	N	Forward 5’- CAAAACCTTCCCTAAGAAGGAAAAG -3’<br>Reverse 5’- GCTCCTTTGGAGGTTCAGACAT -3’<br>Probe 5’-FAM-ACAAAAGGCACCAAAAGAAGAATCAACAGACC-BHQ1-3'
Human	ACE2	Forward 5’- CATTGGAGCAAGTGTTGGATCTT -3’<br>Reverse 5’- GAGCTAATGCATGCCATTCTCA -3’
	GAPDH	Forward 5’- ATTCCACCCATGGCAAATTC -3’<br>Reverse 5’- CGCTCCTGGAAGATGGTGAT -3’
	TMPRSS2	Forward 5’- CTCTACGGACCAAACTTCATC -3’<br>Reverse 5’- CCACTATTCCTTGGCTAGAGTA -3’
	Furin	Forward 5’- CCTGGTTGCTATGGGTGGTAG -3’<br>Reverse 5’- AAGTGGTAATAGTCCCCGAAGA -3’
	Cathepsin L	Forward 5’- GTGGACATCCCTAAGCAGGA -3’<br>Reverse 5’- CACAATGGTTTCTCCGGTC -3’
	Cathepsin B	Forward 5’- AGAGTTATGTTTACCGAGGACCT -3’<br>Reverse 5’- GATGCAGATCCGGTCAGAGA -3’
