# Supplementary material for: Host and viral determinants for efficient SARS-CoV-2 infection of the human lung
Source: Nat Commun. 2021 Jan 8;12:134. doi: 10.1038/s41467-020-20457-w (PMC7794309; doi:10.1038/s41467-020-20457-w)
Supplement: Supplementary file 3 — Reporting Summary [file 41467_2020_20457_MOESM3_ESM.pdf]

nature research

Corresponding author(s): Kwok-Yung Yuen

Last updated by author(s): Nov 28, 2020

Reporting Summary

Nature Research wishes to improve the reproducibility of the work that we publish. This form provides structure for consistency and transparency in reporting. For further information on Nature Research policies, see our Editorial Policies and the Editorial Policy Checklist.

Statistics

For all statistical analyses, confirm that the following items are present in the figure legend, table legend, main text, or Methods section.

n/a	Confirmed	
☐	☒	The exact sample size (*n*) for each experimental group/condition, given as a discrete number and unit of measurement
☐	☒	A statement on whether measurements were taken from distinct samples or whether the same sample was measured repeatedly
☐	☒	The statistical test(s) used AND whether they are one- or two-sided<br>*Only common tests should be described solely by name; describe more complex techniques in the Methods section.*
☒	☐	A description of all covariates tested
☒	☐	A description of any assumptions or corrections, such as tests of normality and adjustment for multiple comparisons
☐	☒	A full description of the statistical parameters including central tendency (e.g. means) or other basic estimates (e.g. regression coefficient) AND variation (e.g. standard deviation) or associated estimates of uncertainty (e.g. confidence intervals)
☐	☒	For null hypothesis testing, the test statistic (e.g. *F*, *t*, *r*) with confidence intervals, effect sizes, degrees of freedom and *P* value noted<br>*Give P values as exact values whenever suitable.*
☒	☐	For Bayesian analysis, information on the choice of priors and Markov chain Monte Carlo settings
☒	☐	For hierarchical and complex designs, identification of the appropriate level for tests and full reporting of outcomes
☒	☐	Estimates of effect sizes (e.g. Cohen's *d*, Pearson's *r*), indicating how they were calculated

Our web collection on statistics for biologists contains articles on many of the points above.

Software and code

Policy information about availability of computer code

Data collection	Olympus cellSens Dimension (version 1.17) was used to collect microscope data.<br>LightCycler96 (version SW1.1) was used to collect qPCR data.<br>Biacore ×100 evaluation software (version 2.0.1) was used to collect the SPR data.<br>Alliance Q9 software (v17-02) was used to collect the Western blot data.
Data analysis	GraphPad Prism (version 6.0) was used for analysis and plotting the figures.<br>FlowJo (version vX.0.7) was used to analyze the flow cytometry data.

For manuscripts utilizing custom algorithms or software that are central to the research but not yet described in published literature, software must be made available to editors and reviewers. We strongly encourage code deposition in a community repository (e.g. GitHub). See the Nature Research guidelines for submitting code & software for further information.

Data

Policy information about availability of data

All manuscripts must include a data availability statement. This statement should provide the following information, where applicable:

- Accession codes, unique identifiers, or web links for publicly available datasets
- A list of figures that have associated raw data
- A description of any restrictions on data availability

The data that support the findings of this study are available from the corresponding authors upon reasonable request.

Field-specific reporting

Please select the one below that is the best fit for your research. If you are not sure, read the appropriate sections before making your selection.

- [x] Life sciences
- [ ] Behavioural & social sciences
- [ ] Ecological, evolutionary & environmental sciences

For a reference copy of the document with all sections, see nature.com/documents/nr-reporting-summary-flat.pdf

Life sciences study design

All studies must disclose on these points even when the disclosure is negative.

Sample size	All experiments were repeated at least three times to give a n number of 3 or above. A n number equals to 3 is the the standard of biological experiments. No sample size calculation was performed. Sample size is chosen based on the standard of the corresponding field.
Data exclusions	No data were excluded.
Replication	All experiments were repeated at least three times on three different days. Similar findings were obtained from all repeats.
Randomization	Randomization is not relevant to our study. In our study, the experiments are well-controlled and the same number of cells are used for comparison so that there is no background difference between experimental groups.
Blinding	Blinding is not relevant to our study. The experiments for different groups are carried out in parallel using the same set of protocols and the experimental results are quantitative.

Reporting for specific materials, systems and methods

We require information from authors about some types of materials, experimental systems and methods used in many studies. Here, indicate whether each material, system or method listed is relevant to your study. If you are not sure if a list item applies to your research, read the appropriate section before selecting a response.

Materials & experimental systems

n/a	Involved in the study	
[ ]	[x]	Antibodies
[ ]	[x]	Eukaryotic cell lines
[x]	[ ]	Palaeontology and archaeology
[x]	[ ]	Animals and other organisms
[ ]	[x]	Human research participants
[x]	[ ]	Clinical data
[x]	[ ]	Dual use research of concern

Methods

n/a	Involved in the study	
[x]	[ ]	ChIP-seq
[ ]	[x]	Flow cytometry
[x]	[ ]	MRI-based neuroimaging

Antibodies

Antibodies used	Rabbit anti-human furin (#ab183495, Abcam) 1:5000 for Western blots;<br>Rabbit anti-V5 (MAB8926, R&D) 1:2500 for Western blots;<br>Mouse anti-human beta-actin antibody (#MAB8929, R&D) 1:5000 for Western blots;<br>In-house rabbit anti-SARS-CoV-2-nucleocapsid (N) immune serum 1:3000 for immunofluorescence staining, 1:10000 for Western blots;<br>In-house rabbit anti-SARS-CoV-N immune serum 1:3000 for immunofluorescence staining;<br>Rabbit anti-SARS-CoV-2 spike S2 (40590-T62, Sino Biological) 1:5000 for Western blots;<br>Goat anti-human ACE2 (AF933, R&D Systems) 1.35ug/mL for flow cytometry;<br>Donkey anti-Goat IgG (H+L), Alexa Fluor 488 (A11055, Thermo Fisher Scientific) 1:300 for flow cytometry.<br>Goat anti-Rabbit IgG (H+L), Alexa Fluor 488 (A11034, Thermo Fisher Scientific) 1:4000 for immunofluorescence staining.<br>Goat anti-Rabbit IgG (H+L) HRP (31460, Thermo Fisher Scientific) 1:5000 for Western blots;<br>Goat anti-Mouse IgG (H+L) HRP (31430, Thermo Fisher Scientific) 1:5000 for Western blots;
Validation	Commercial primary antibodies were validated by the manufacturers and validation statements are available on the manufacturer's website.<br>The in-house anti-SARS-CoV-2-N and anti-SARS-CoV-N immune serum were validated with ELISA, Western blots, and immunofluorescence staining in our previous publication (PMID: 32835326).

Eukaryotic cell lines

Policy information about cell lines

Cell line source(s)	Calu3, Caco2, Huh7, BHK21, RK13, PK15, CRFK, 293T, and VeroE6 were obtained from ATCC.
Authentication	The cell lines were not authenticated.
Mycoplasma contamination	All cell lines have been recently tested negative for mycoplasma contamination.
Commonly misidentified lines (See ICLAC register)	No commonly misidentified cell lines were used in this study.

Human research participants

Policy information about studies involving human research participants

Population characteristics	A total of 9 patient donors with lung tumour who underwent wedge resection or lobectomy were included in this study. These included 3 females and 6 males with a mean age of 65.8 years (range, 57-80 years)<br>A total of 9 patients donors with large intestine tumour who underwent surgery were included in this study. These included 4 female and 5 males with a mean age of 69 years (range, 54-82 years).
Recruitment	Human lung and intestine tissues for ex vivo studies were obtained from patients undergoing surgical operations at Queen Mary Hospital, Hong Kong. All donors gave written consent. There is no potential selection bias.
Ethics oversight	The study protocol is approved by the Institutional Review Board of the University of Hong Kong/Hospital Authority Hong Kong West Cluster (UW13-364).

Note that full information on the approval of the study protocol must also be provided in the manuscript.

Flow Cytometry

Plots

Confirm that:

- [x] The axis labels state the marker and fluorochrome used (e.g. CD4-FITC).
- [x] The axis scales are clearly visible. Include numbers along axes only for bottom left plot of group (a 'group' is an analysis of identical markers).
- [x] All plots are contour plots with outliers or pseudocolor plots.
- [x] A numerical value for number of cells or percentage (with statistics) is provided.

Methodology

Sample preparation	BHK21 cells were detached from the culture plate with 10mM EDTA and fixed in 4% paraformaldehyde.
Instrument	BD FACSCanto II cell analyzer was used for data collection.
Software	FlowJo X 10.0.7 was used for data analysis.
Cell population abundance	Not applicable. Did not sort cells.
Gating strategy	The main BHK21 cell population was gated with SSC-A vs FSC-A. The selected cells were gated with FSC-H vs FSC-A for single cells. From that, ACE2 positive cells were gated with FSC-H vs FITC-A. BHK21 cells labeled with isotype control and secondary antibody were used as the control for gating. The gating strategy was demonstrated in Figure S3.

- [x] Tick this box to confirm that a figure exemplifying the gating strategy is provided in the Supplementary Information.
